# Dynamic Alterations in Yak Rumen Bacteria Community and Metabolome Characteristics in Response to Feed Type

**DOI:** 10.3389/fmicb.2019.01116

**Published:** 2019-05-22

**Authors:** Chang Liu, Hao Wu, Shujie Liu, Shatuo Chai, Qingxiang Meng, Zhenming Zhou

**Affiliations:** ^1^State Key Laboratory of Animal Nutrition, College of Animal Science and Technology, China Agricultural University, Beijing, China; ^2^Qinghai Academy of Animal and Veterinary Sciences, Qinghai University, Xining, China

**Keywords:** yak, rumen, microbiota, metabolomics, feed type

## Abstract

Current knowledge about the relationships between ruminal bacterial communities and metabolite profiles in the yak rumen is limited. This is due to differences in the nutritional and metabolic features between yak and other ordinary cattle combined with difficulties associated with farm-based research and a lack of technical guidance. A comprehensive analysis of the composition and alterations in ruminal metabolites is required to advance the development of modern yak husbandry. In the current study, we characterized the effect of feed type on the ruminal fluid microbiota and metabolites in yak using 16S rRNA gene sequencing and liquid chromatography-mass spectrometry (LC-MS). *Bacteroidetes* and *Firmicutes* were the predominant bacterial phyla in the yak rumen. At the genus level, the relative abundance of *Bacteroidales BS11 gut group, Prevotellaceae UCG-003, Ruminococcaceae UCG-011, Bacteroidales RF16 group* and *Ruminococcaceae UCG-010* was significantly (*P* < 0.01) higher in the forage group compared to that in the concentrate group, while the concentrate group harbored higher proportions of *Bacteroidales S24-7 group, Ruminococcaceae NK4A214, Succiniclasticum* and *Ruminococcus 2*. Yak rumen metabolomics analysis combined with enrichment analysis revealed that feed type altered the concentrations of ruminal metabolites as well as the metabolic pattern, and significantly (*P* < 0.01) affected the concentrations of ruminal metabolites involved in protein digestion and absorption (e.g., L-arginine, ornithine, L-threonine, L-proline and β-alanine), purine metabolism (e.g., xanthine, hypoxanthine, deoxyadenosine and deoxyadenosine monophosphate) and fatty acid biosynthesis (e.g., stearic acid, myristic acid and arachidonic acid). Correlation analysis of the association of microorganisms with metabolite features provides us with a comprehensive understanding of the composition and function of microbial communities. Associations between utilization or production were widely identified between affected microbiota and certain metabolites, and these findings will contribute to the direction of future research in yak.

## Introduction

The yak (*Bos grunniens*), which is an iconic symbol of the Qinghai-Tibetan plateau, thrives in extraordinarily harsh conditions and provides the basic resources for nomadic pastoralists. For centuries, the yak has been inextricably linked with human civilization and survival in such a hostile environment (Wiener et al., 2003). Although many technical developments and management strategies have led to advances in yak husbandry, many nomadic pastoralists are still heavily influenced by traditional values. The traditional approach to grazing management is largely dependent on natural pastures (Long et al., 1999) and limited supplementary feeding regimes during periods of limited food availability (Ding et al., 2015). Therefore, nutritional management, especially the choice of feed type, is extremely important to improve farmed yak performance.

Rumen function has a critical impact on ruminant production systems. Bacteria, which are the most abundant, diverse and metabolically active ruminal microbes, enable the ruminant to ferment plant proteins and polysaccharides to generate the nutrients necessary for maintenance and growth (Deusch et al., 2017; Seshadri et al., 2018). However, a wide range of metabolites that perform a vast array of complex metabolic activities in the rumen are also used by the microbes for their own proliferation (Saleem et al., 2013). Previous studies have revealed interactions between the diversity of functional classes or unique niches of microorganisms and the metabolic factors that govern microbial metabolism and associated nutrient formation pathways (Bannink et al., 2016). Furthermore, the rumen microbiome has been shown to be dramatically affected by changes in feeding strategies and diets (Mohammed et al., 2014; Henderson et al., 2015; Zhang J. et al., 2017) and also to exhibit host individuality (Weimer et al., 2010; Weimer, 2015).

A comprehensive analysis of the composition and dynamics of the yak rumen microbiome will offer important insights into microbially-mediated metabolic processes and help improve the efficiency and effectiveness of farming and veterinary practices (Zhang R. et al., 2017). Such insights are also crucial to the development of technologies that support modern yak husbandry.

16S rRNA sequencing technology is a well-tested, fast and cost-effective method for analysis of differential abundance of microbial communities and correlations with environment factors (Jami et al., 2013; Henderson et al., 2015; Lin et al., 2015; Paz et al., 2016; McGovern et al., 2018). Metabolomics analyses based on techniques such as liquid chromatography-mass spectrometry (LC-MS) and gas chromatography-mass spectrometry are valuable emerging tools for targeted and non-targeted profiling of a vast number of small molecular metabolites (amino acids, lipids, organic acids, nucleotides, etc.) in biological samples (biofluids or tissues). These key technologies are also invaluable in studies of influences of environmental influences, disease, or genotype on the metabolomic phenotype (Boudonck et al., 2009; Saleem et al., 2013; Artegoitia et al., 2017; Saro et al., 2018). Of the two methods, gas chromatography-mass spectrometry achieves better metabolite separation than LC-MS but requires chemical derivatization of the metabolic species prior to the analysis. In contrast, LC-MS is capable of detecting a larger pool of intact metabolites with no need for chemical modification (Dörmann, 2000; Gowda and Djukovic, 2014). Furthermore, mass spectrometry coupled to liquid chromatography is an essential technology for non-targeted metabolite profiling studies that aim to provide a comprehensive analysis of all detectable compounds without any prior knowledge (Dettmer et al., 2010; Neumann et al., 2013).

We hypothesized that different feed types (concentrate and forage) would affect the ruminal microbiota and metabolites. However, as yet, there are no reports about the relationships between ruminal bacterial communities and metabolite profiles in the yak rumen. This limitation is due to differences in the nutritional and metabolic features between yak and other ordinary cattle combined with difficulties associated with farm-based research and the lack of technical guidance. In this study, we characterized the yak ruminal fluid microbiota and metabolites to determine the effect of two different feed types on the profiles of the ruminal microbiota and metabolites in yak through a combination of the 16S rRNA gene sequencing and LC-MS. We also explored the possible relationships between ruminal microbiota and metabolites.

## Methods

### Animals, Feedstuffs, and Feeding Regimes

Sixteen male yaks (195 ± 50 kg live weight, aged 4 years) were selected and randomly divided into four groups, and sixteen different feedstuffs (shown in the Supplementary Table S2) were analyzed individually using the yaks. To be specific, we conducted four 4 × 4 Latin square trials, within each time four different feedstuffs were used. The feeding regimes were shown in the Supplementary Table S1. Each phase of the Latin square trail lasted 22 days, of which the first 15 days were for adaptation and the last day (day 22) was for sampling. Subsequently, we divided the feeds into two groups: Concentrate Group (C) and Forage Group (F) according to the crude fiber content. In this study, we defined Forage Group with crude fiber content of 25 to 45% (F), which included wheat straw, pea stem, broad bean stem, rapeseed straw, oat straw and alfalfa. We define Concentrate Group (C) with crude fiber content below 18%, which included soybean meal, broad bean, rape cake, sesame cake, oat, hulless barley, corn, barley, wheat, and wheat bran. Feeds from Forage Group were fed alone, however, feeds from Concentrate Group were fed as pellets (processing pore size was 6 mm) alone with 30% oat straw. In order to maintain normal fermentation in the rumen, the following additives were added into the diets on a DM basis: 2% rumen buffer (NaHCO_3_:MgO ratio 2: 1), 0.5% limestone, 0.5% salt, and 30 mg/kg rumensin. The procedures for the care and use of animals in this study were approved and conducted according to standards established by the College of Animal Science and Technology, CAU, Beijing, P. R. China (permit number DK1402006). The steers were fed the experimental diets at 1.9% BW on a dry matter basis in two equal portions at 0800 and 1600.

### Rumen Sample Collection and Measurements

At the end of each phase in the experiment (day 22), ruminal fluid was collected before morning feeding by introducing a flexible oral stomach tube containing a metal strainer into the rumen and using the suction created with a 50-mL syringe to remove the fluid from the tube. The device was cleaned thoroughly between sample collections using fresh warm water, and the first 50-mL of rumen fluid was discarded to minimize saliva contamination. Then, 50 mL of rumen fluid was collected from each animal and transferred into a separate sterilized container, immediately frozen with liquid nitrogen, and stored at−80°C prior to processing. As a result, a total of 64 yak rumen fluid samples should be collected in this study, but unfortunately 6 rumen fluid samples (three of them from the concentrate group and another three from the forage group) were damaged, so the analysis was performed using the left 58 rumen fluid samples. Rumen pH was measured after collection using a portable pH meter (Testo205; Testo AG, Schwarzwald, Germany). Filtered rumen fluid was centrifuged at 8,000 × g and 4°C for 15 min) to obtain the supernatant, which was further used to determine the concentrations of volatile fatty acids (VFA) and ammonia-nitrogen (NH_3_-N). The VFA profile was determined with GC 3420 gas chromatography fitted with HP-INNO wax capillary column (30 m × 0.32 mm; Erwin et al., 1961), and NH_3_-N concentration was analyzed by visible spectrophotometry(UV-VIS8500, Tianmei, Shanghai, China; Broderick and Kang, 1980).

### DNA Extraction, 16S rRNA Gene Amplification, and Sequencing

Microbial DNA was extracted from 58 rumen fluid samples using the E.Z.N.A.® soil DNA Kit (Omega Bio-tek, Norcross, GA, USA) according to the manufacturer's protocols. The DNA concentration and purity were determined by NanoDrop 2000 UV-vis spectrophotometer (Thermo Scientific, Wilmington, NC, USA), and DNA quality was evaluated by 1% agarose gel electrophoresis. The V3 and V4 hypervariable regions of the bacterial 16S rRNA gene were amplified with primers 338F (5′- ACTCCTACGGGAGGCAGCAG-3′) and 806R (5′-GGACTACHVGGGTWTCTAAT-3′) using a thermocycler PCR system (GeneAmp 9700, ABI, USA). The PCR reactions were performed in triplicate in a total reaction volume of 20 μL containing 4 μL of 5 × FastPfu Buffer, 2 μL of 2.5 mM dNTPs, 0.8 μL of Forward Primer (5 μM) and Reverse primer (5 μM), 0.4 μL of FastPfu Polymerase, 0.2 μL of BSA and 10 ng of template DNA. The amplified products were detected using 2% agarose gel electrophoresis, further purified using the AxyPrep DNA Gel Extraction Kit (Axygen Biosciences, Union City, CA, USA) and then quantified using QuantiFluor™-ST (Promega, USA) according to the manufacturers' protocols. Following amplification, paired-end sequencing libraries were constructed by Majorbio Bio-Pharm Technology Co. Ltd. (Shanghai, China). Subsequently, purified amplicons were pooled in equimolar amounts and sequenced on an Illumina MiSeq platform (Illumina, San Diego, CA, USA) for paired-end reads of 300 bp at Majorbio Bio-Pharm Technology Co. Ltd. (Shanghai, China) according to standard protocols.

### Sequence Processing and Analysis

Raw FASTQ files were quality-filtered by Trimmomatic and merged by FLASH according to the following criteria: (i) The reads were truncated at any site receiving an average quality score < 20 over a 50-bp sliding window; (ii) Sequences with overlaps longer than 10-bp were merged according to their overlap with mismatches ≤ 2 bp; (iii) Sequences of each sample were separated according to barcodes (exactly matching) and primers (allowing 2 nucleotide mismatches), and reads containing ambiguous bases were removed. Operational taxonomic units (OTUs) were clustered with a 97% similarity cut-off using UPARSE (version 7.1 http://drive5.com/uparse/) with a novel “greedy” algorithm that performs chimera-filtering and OTU-clustering simultaneously (Edgar, 2013). The taxonomy of each 16S rRNA gene sequence was analyzed using the RDP Classifier algorithm (http://rdp.cme.msu.edu/) against the Silva (SSU123) 16S rRNA database with a confidence threshold of 70% (Wang et al., 2007; Quast et al., 2013).

Analysis was performed using the free online platform, Majorbio I-Sanger Cloud Platform (http://www.i-sanger.com). Alpha diversity indexes were calculated using MOTHUR (version v.1.30.1) (Schloss et al., 2009). The rarefaction curve and bar graphs were generated using vegan package in R (Oksanen et al., 2010). Beta-diversity was estimated by computing the unweighted UniFrac distance and visualized using principal coordinate analysis (PCoA), and the results were plotted using GUniFrac and ape packages in R (Chen et al., 2012; Paradis and Schliep, 2018). The Wilcoxon rank-sum test within STAMP (version v.2.1.3) was used to identify phyla and genera that showed significant differences in abundance between groups (confidence interval method) with the Stats package in R and the SciPy package in PYTHON (Jones et al., 2001; R Core Team, 2013; Parks et al., 2014).

### LC-MS Metabolomics Processing

A total of 58 rumen samples were analyzed using the LC-MS platform (Thermo, Ultimate 3000LC, Q Exactive). After samples were thawed at room temperature, 100 μL of each sample was then transferred into centrifuge tubes (1.5 mL) by pipette and 300 μL of methanol was added. After the addition of 10 μL of internal standard (3.0 mg/mL, DL-o-chlorophenylalanine), each sample was vortexed for 30 s, and then centrifuged at 12,000 rpm for 15 min at 4°C. Next, 200 μL of the supernatant was transferred to a new vial for LC-MS analysis. Chromatographic separation was performed using a Hyper gold C18 column (100 × 4.6 mm, 3 μm internal diameter) preheated to 40°C. A prepared rumen sample of 10 μL was injected and maintained at 4°C for analysis. Samples were eluted using a mobile phase for positive ion mode (ESI+) and negative ion mode (ESI–) composed of water and 5% acetonitrile with 0.1% formic acid as solvent A, and acetonitrile with 0.1% formic acid as solvent B, at a flow rate was at 0.35 mL/min and the following mobile phase (A:B) elution gradient: 100%:0% for 0–1.5 min, 80%:20% at 1.5 min, and 0%:100 % at 9.5 min followed by 3 min of re-equilibration. The ion source temperature was 300°C, and the capillary temperature was 350°C. The flow rate of Sheath Gas, Aux Gas and Sweep Gas was 45 arb, 15 arb, and 1 arb, respectively. The spray voltage was 3.0 kV and 3.2 kV for the positive and negative mode, respectively. The S-Lens RF level was set at 60%. To obtain information regarding system repeatability, quality control (QC) samples prepared by mixing all rumen fluid extraction aliquots were injected at regular intervals throughout the analytical run.

### Metabolomics Data Analysis

The data were first transformed to CDF files by Thermo Scientific™ Xcalibur™ (version v3.0). Peak picking, peak alignment, peak filtering, and peak filling were performed using XCMS software (version v.3.4.4). The data for retention time (RT), MZ, observations (samples) and peak intensity were normalized using Excel. The positive and negative data were imported into the SIMCA-P software package. Principle component analysis (PCA) and (orthogonal) partial least squares discriminant analysis (O)PLS-DA were carried out to visualize the metabolic alterations among experimental groups, after mean centering and unit variance scaling. Variable importance in the projection (VIP) ranks the overall contribution of each variable to the PLS-DA model, and those variables with VIP >1.0 are considered relevant for group discrimination. In this study, the default 7-round cross-validation was applied with one-seventh of the samples being excluded from the mathematical model in each round to guard against overfitting.

Significant differences in metabolites between groups were analyzed using a combination of (O)PLS-DA and Student's *t*-tests, with *P*-values < 0.05 considered to indicate statistical significance. Screened differential metabolites were characterized using the https://metlin.scripps.edu/ public database and a self-built database of the Majorbio I-Sanger Cloud Platform (https://www.i-sanger.com). Significantly differentially expressed metabolites were analyzed for expression pattern clustering using the gplots package in R (Warnes et al., 2016). The following distance calculation algorithms were used: Spearman between samples, Pearson between metabolites, and clustering method for H cluster (complete algorithm). The impacts of feed type on metabolic pathways and metabolite set enrichment were analyzed using the Stats package in R and the SciPy package in PYTHON (Jones et al., 2001; R Core Team, 2013). The results were tested using Fisher's exact test in R. Correlations between different metabolites and bacterial communities were assessed by Spearman's correlation analysis using the pheatmap package in R (Kolde, 2012). *P*-values were adjusted with FDR and the corrected *P*-values below 0.05 were regarded as statistically significant.

## Results

### Rumen Fermentation Parameters

The fermentation characteristics (pH, NH_3_-N and VFA profile) are showed in Table 1. In general, the type of feedstuff significantly affected (*P* < 0.05) the NH_3_-N concentration, VFA production and the proportions of acetate, isobutyrate, butyrate, isovalerate, and valerate. Among them, the concentrate group contained high levels of NH_3_-N and the proportions of isobutyrate, butyrate, isovalerate and valerate. Meanwhile, higher VFA production and the proportion of acetate were observed in the forage group. However, the proportion of propionate and ratio of acetate: propionate showed no significant differences between the concentrate group and the forage group (*P* > 0.05). The pH value of the concentrate group and the forage group was 7.64 and 7.71, respectively.

**Table 1:** Rumen fermentation parameters affected by different feed types.

**Items**	**C**	**F**	**SEM**	***P*-value**
pH	7.64	7.71	0.038	0.343
NH_3_-N, mg/100 mL	9.87	6.26	0.855	0.039
TVFA, mmol/L	47.66	58.74	1.944	0.0046
**VFA, %**				
Acetate, %	71.45	75.45	0.004	<0.0001
Propionate, %	15.49	16.6	0.003	0.0581
Isobutyrate, %	1.43	0.62	0.00089	<0.0001
Butyrate, %	8.82	5.96	0.003	<0.0001
Isovalerate, %	2.24	1.15	0.00098	<0.0001
Valerate, %	0.56	0.22	0.0003	<0.0001
Acetate: Propionate	4.73	4.58	0.0902	0.4314

*C, the concentrate group; F, the forage group; SEM, standard error of the mean*.

### Richness, Diversity Estimates, and Rumen Bacteria Composition

In total, 3,223,324 high-quality 16S rRNA gene sequences were obtained from 58 samples. After sub-sampling each sample to an equal sequencing depth (44,760 reads per sample) and clustering, we obtained 3,149 OTUs at 97% identity. Rarefaction curves were generated using observed OTUs for each feed type to assess the adequacy of the sampling depth to evaluate rumen bacterial composition. Rarefaction curves showed a diminishing rate of new OTU identification as the number of reads per sample increased, implying that the sampling depth was adequate for evaluating dominant members of the rumen bacterial community. Similarly, the Good's coverages for all samples exceeded 98%, which indicated the accuracy and reproducibility of the sequencing.

**Figure 1:**
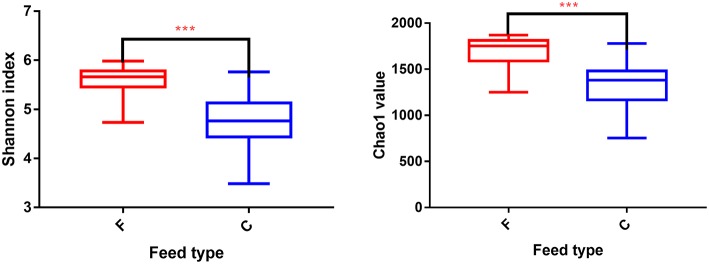
Differences in Yak ruminal bacterial diversity and richness between the concentrate and forage groups. Bacterial diversity was estimated by Shannon index. Bacterial richness estimated by the Chao1 value. C, concentrate group; F, forage group. ***indicate significant difference between the Concentrate Group and the Forage Group (*P* ≤ 0.001).

**Figure 2:**
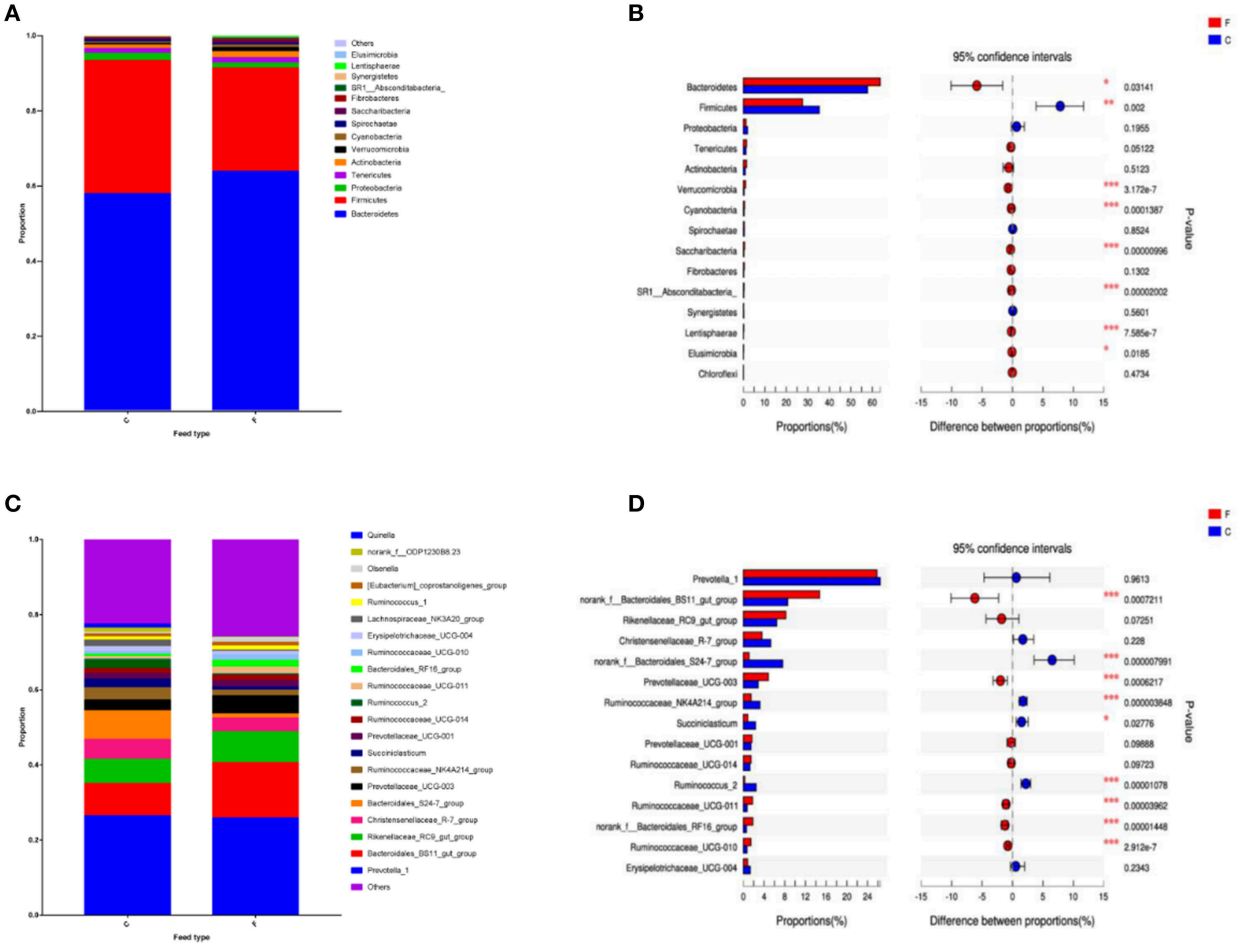
Classification of the bacterial community composition across the forage and concentrate groups. **(A)** Phylum level. **(B)** Extended error bar plot showing the bacteria at the phylum level that had significant differences between the concentrate and forage groups. **(C)** Genus level. **(D)** Extended error bar plot showing the bacteria at the genus level that had significant differences between the concentrate and forage groups. Positive differences indicate greater abundance of bacteria at the phylum level and at the genus level in the concentrate group, while negative differences indicate greater abundance in the forage group. C, concentrate group; F, forage group. Asterisks indicate significant difference between the Concentrate Group and the Forage Group (*0.01 < *P* ≤ 0.05; **0.001 < *P* ≤ 0.01; ***P ≤ 0.001).

According to the Shannon index (5.584 ± 0.285 vs. 4.778 ± 0.506, *P* < 0.01) and Chao1 value (1685.67 ± 136.52 vs. 1336.82 ± 230.72, *P* < 0.01), there were significant differences in microbiota diversity and richness between the two groups, indicating higher diversity in the forage group and less richness in the concentrate group (Figure 1). Taxonomic analysis of the reads revealed the presence of 23 bacterial phyla, with *Bacteroidetes* and *Firmicutes* being the predominant phyla accounting for 59.75% and 32.70% of the total reads, respectively (Figure 2A). At the genus level, 336 genera were identified in the yak rumen samples. The predominant genera were *Prevotella 1* (26.21%), *Bacteroidales BS11 gut group* (10.81%), *Rikenellaceae RC9 gut group* (7.09%), *Bacteroidales S24-7 group* (5.26%), *Christensenellaceae R-7 group* (4.69%), *Prevotellaceae UCG-003* (3.58%) *and Ruminococcaceae NK4A214 group* (2.61%), respectively (Figure 2C).

### Differences in Bacterial Community Composition Between the Two Feed Types

**Figure 3:**
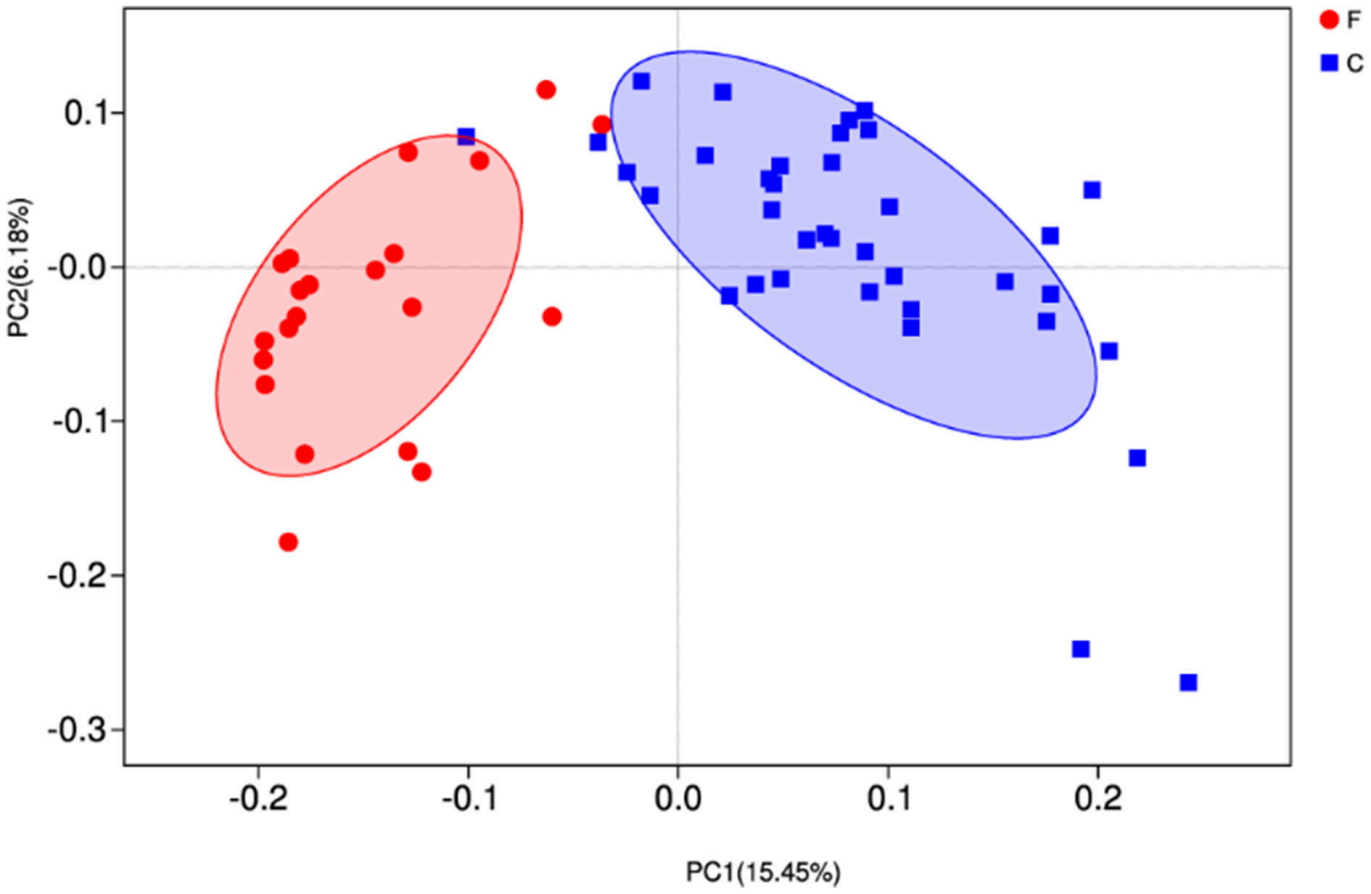
Principal coordinate analysis (PCoA) of rumen microbial communities. C, concentrate group; F, forage group.

At phylum level (Figure 2B), the relative abundances of *Bacteroidetes, Verrucomicrobia, Cyanobacteria, Saccharibacteria, SR1 Absconditabacteria, Lentisphaerae*, and *Elusimicrobia* were significantly higher in the forage group (*P* < 0.05) compared to those in the concentrate group, while the ruminal microbiome of the concentrate group had a higher abundance of *Firmicutes* compared to the forage group. Genus level (Figure 2D) classification of bacterial communities within the two different feed types showed significantly (*P* < 0.01) higher abundances of *Bacteroidales BS11 gut group, Prevotellaceae UCG-003, Ruminococcaceae UCG-011, Bacteroidales RF16 group* and *Ruminococcaceae UCG-010* in the forage group compared to those in the concentrate group. On the other hand, the relative abundances of *Bacteroidales S24-7 group, Ruminococcaceae NK4A214, Succiniclasticum* and *Ruminococcus 2* were higher in the concentrate group than those in the forage group. Furthermore, PCoA plots using the unweighted UniFrac matrix distances, where bacterial communities clustered by feedstuff type, clearly showed the distinct bacterial community structure in the concentrate and forage groups (Figure 3), indicating that the feed type influences the bacterial community composition.

### Correlations Between Rumen Bacteria and Rumen Fermentation Parameters

**Figure 4:**
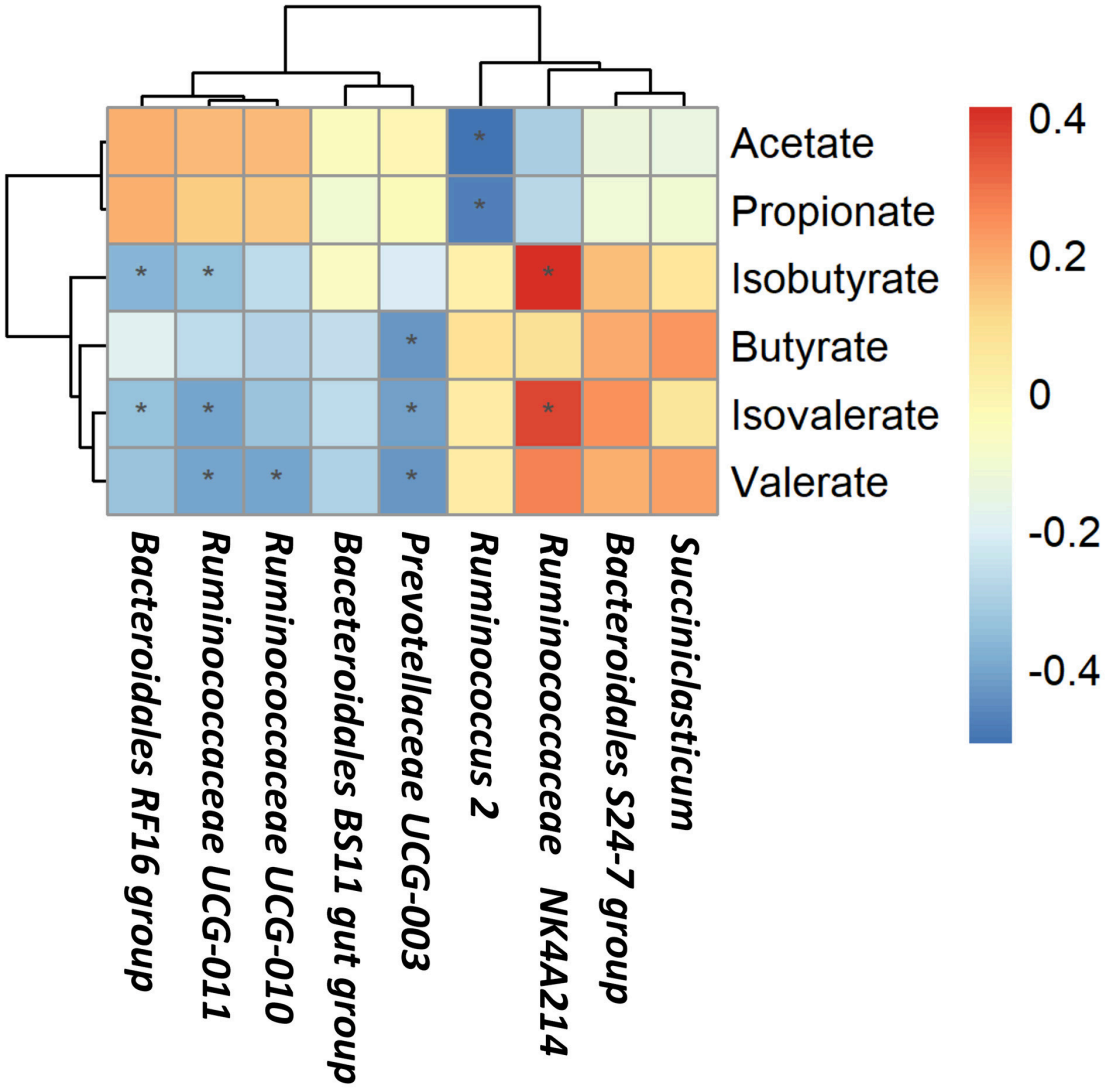
Correlations between rumen bacteria and rumen fermentation parameters. Each row in the graph represents a genus, each column represents a metabolite, and each lattice represents a Pearson correlation coefficient between a component and a metabolite. Red represents a positive correlation, while blue represents a negative correlation. *Significant correlation between the concentrate and forage groups (*P* < 0.05).

Based on Spearman's correlation coefficients, the significantly affected ruminal microbiota (at the genera level) appeared to be significant correlated with rumen fermentation parameters (Figure 4). *Ruminococcus 2* was negatively correlated with acetate and propionate concentrations; *Bacteroidales RF16 group* and *Ruminococcaceae UCG-011* were negatively correlated with isobutyrate and isovalerate concentrations while *Ruminococcaceae NK4A214 group* was positively correlated with isobutyrate and isovalerate concentrations; *Ruminococcaceae UCG-011* and *Ruminococcaceae UCG-010* were negatively correlated with valerate concentrations; *Prevotellaceae UCG-003* was negatively correlated with butyrate, valerate, and isovalerate concentrations.

### Rumen Metabolomics Profiling

Supplementary Figure S1 show the overlap of the total ion chromatogram of the QC sample in the positive (A) and negative (B) ion modes, respectively. The results confirm the reliable repeatability and precision of the data obtained in this study.

**Figure 5:**
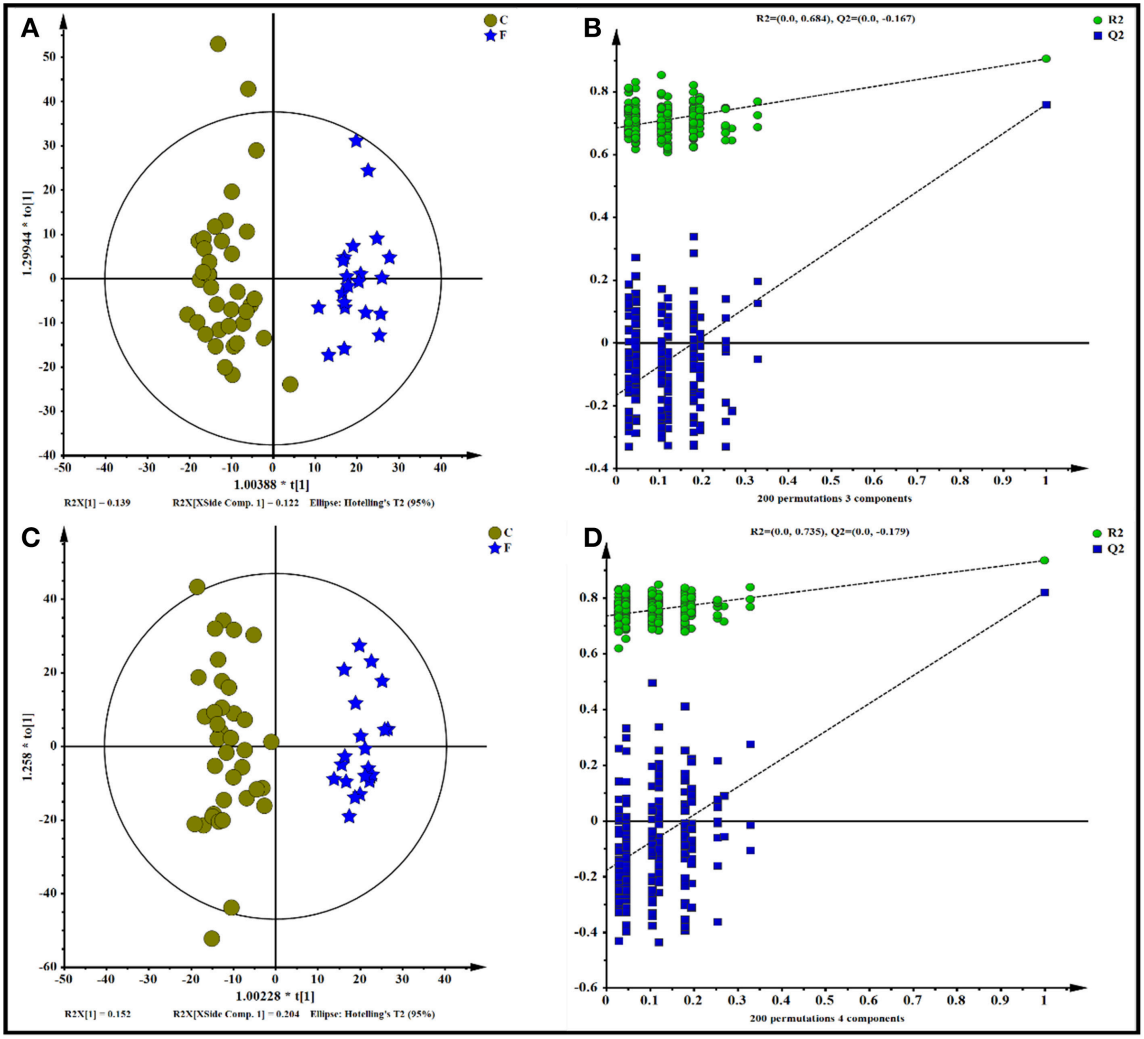
Orthogonal partial least squares discriminant analysis [(O)PLS-DA] plot of yak rumen metabolites in comparisons of the concentrate and forage groups following **(A,B)** positive and **(C,D)** negative mode ionization.

All data, including the QC samples that were included throughout the analysis, were first examined by PCA following positive and negative mode ionization (Supplementary Figures S2A,B, respectively) to provide a global overview of the differences among the metabolite data. Score plots of the (O)PLS-DA performed to verify the differentiated metabolites between the two groups and supervise the multivariate analysis are shown in Figure 5. The (O)PLS-DA provides valuable insights into group relationships from simple visual inspection of scores-space clustering patterns. All the samples in the score plots were within the 95% Hotelling T2 ellipse, whereas only two samples of the yak rumen fluid were outside the ellipse. For the positive ionization analysis, the (O)PLS-DA fitted model (Figure 5A) resulted in one predictive and two orthogonal components. Furthermore, 32.3% of the total explained variation in the data set (R^2^X cum) was used to account for 90.4% of the variance in the class separation (R^2^Y cum), and the cross-validated predictive ability of the model was 77.5% (Q^2^ cum). As shown in Figure 5B, the R^2^Y value (0.684) and the Q^2^Y value (−0.167 < 0) of the permutation test indicated satisfactory effectiveness of the model. The results of the (O)PLS-DA results and permutation tests following negative mode ionization are shown in Figures 5C,D. Both positive and negative data revealed clear separation and discrimination between the concentrate and forage groups, indicating that the (O)PLS-DA model can be used to identify differences between the two groups.

As shown in Table 2 and Supplementary Table S3, 87 differential metabolites (64 positively ionized metabolites and 35 negatively ionized metabolites) between the concentrate and the forage groups were identified using a VIP threshold of 1 (*P* < 0.05). Among 64 positively ionized metabolites, 24 were classified as lipids and lipid-like molecules, 14 as organic acids and derivatives, nine as organoheterocyclic compounds, five as nucleosides, nucleotides, and analogs, four as organic oxygen compounds, four as organic nitrogen compounds, and four as benzenoids. In the negative ionization analysis, 35 differential metabolites were classified as lipids and lipid-like molecules, organic acids and derivatives, organoheterocyclic compounds, benzenoids, organic nitrogen compounds and nucleosides, nucleotides, and analogs.

**Table 2:** Identification of significant differential metabolites in yak rumen fluid by comparison of the concentrate and forage groups using a VIP threshold of 1 (*P* < 0.05).

**Metabolite name**	**VIP**	**RT (min)**	**Mass error (ppm)**	**Ion (m/z)**	**Fold change**	***p* value**	**Positive/negative**
**AMINO ACIDS, PEPTIDES, AND ANALOGS**							
β-Alanine	1.57953	0.90	27	90.06	1.36	<0.05	ESI+
Ornithine	1.12637	0.82	15	155.08	1.67	<0.05	ESI+
N6,N6,N6-Trimethyl-L-lysine	1.19657	8.52	15	189.16	1.37	<0.05	ESI+
L-Valine	1.84756	10.15	4	140.07	0.88	<0.05	ESI+
L-Tyrosine	1.67342	0.99	18	182.08	1.30	<0.05	ESI+
L-Proline	1.73136	0.84	0	116.07	1.08	<0.05	ESI+
L-Phenylalanine	1.90725	1.54	2	166.09	2.98	<0.05	ESI+
L-Methionine	1.67356	0.97	0	150.06	1.74	<0.05	ESI+
L-Leucine	1.86254	1.13	20	132.10	2.63	<0.05	ESI+
L-Glutamate	1.78202	0.87	11	148.06	1.19	<0.05	ESI+
L-Dopa	1.59629	5.45	15	198.07	−0.25	<0.05	ESI+
L-Arginine	1.12843	6.87	35	197.09	−0.15	<0.05	ESI+
Pyroglutamic acid	1.4513	0.94	12	128.03	2.06	<0.05	ESI−
Citrulline	1.00457	3.13	25	174.08	−0.34	<0.05	ESI−
L-Aspartic acid	1.02264	0.02	24	132.03	2.09	<0.05	ESI−
L-Threonine	1.18916	2.84	35	154.02	−2.19	<0.05	ESI−
**FATTY ACIDS AND CONJUGATES**							
L-Palmitoylcarnitine	1.35173	10.22	18	400.35	1.22	<0.05	ESI+
β-Hydroxyisovaleric acid	1.34658	2.45	22	119.07	−0.19	<0.05	ESI+
Stearic acid	1.88003	10.73	2	283.26	2.68	<0.05	ESI−
Oleic acid	1.71181	10.22	5	283.26	2.20	<0.05	ESI+
Myristoleic acid	1.58974	4.93	14	249.18	−0.94	<0.05	ESI+
Myristic acid	1.16514	5.99	12	251.20	−1.37	<0.05	ESI+
**FATTY ACIDS AND CONJUGATES**							
Eicosadienoic acid	1.1918	6.44	10	331.26	−0.60	<0.05	ESI+
Arachidonic acid	2.26453	6.37	3	305.25	−1.87	<0.05	ESI+
Tetradecanedioic acid	1.34647	5.99	2	257.18	−1.19	<0.05	ESI−
Palmitic acid	1.54094	10.05	3	255.23	1.82	<0.05	ESI−
Adrenic acid	1.76404	9.68	12	367.25	2.32	<0.05	ESI−
**PURINES AND PURINE DERIVATIVES**							
Xanthine	1.61396	0.99	16	153.04	4.64	<0.05	ESI+
Hypoxanthine	2.07019	0.98	4	137.05	3.83	<0.05	ESI+
Guanine	1.63166	0.98	15	152.05	1.74	<0.05	ESI+
Adenine	1.52322	0.85	2	136.06	1.89	<0.05	ESI+
**NUCLEOSIDES, NUCLEOTIDES, AND ANALOGS**							
Deoxyadenosine monophosphate	1.33723	4.47	13	332.08	0.74	<0.05	ESI+
Adenosine monophosphate	1.17168	0.96	8	348.07	1.56	<0.05	ESI+
Inosine	1.67438	0.79	2	291.07	3.29	<0.05	ESI+
Guanosine	1.63239	1.01	1	284.10	2.33	<0.05	ESI+
Deoxyadenosine	1.16584	3.61	9	274.09	−1.24	<0.05	ESI+
Flavin mononucleotide	1.96572	2.60	0	455.10	1.76	<0.05	ESI−
**OTHER LIPIDS AND LIPID-LIKE MOLECULES**							
Linoleic acid	1.3075	8.51	5	281.25	1.93	<0.05	ESI+
9-HODE	1.48486	8.16	4	297.24	0.93	<0.05	ESI+
9-HOTE	1.01731	5.70	5	295.23	0.45	<0.05	ESI+
Taurallocholic acid	1.11887	3.78	24	516.31	−0.14	<0.05	ESI+
Glycoursodeoxycholic acid	1.60604	3.69	38	472.29	−0.22	<0.05	ESI+
**OTHER LIPIDS AND LIPID-LIKE MOLECULES**							
Glycocholic acid	1.04578	9.04	25	466.33	2.49	<0.05	ESI+
LysoPC(16:0)	1.06905	7.07	2	518.32	2.63	<0.05	ESI+
LysoPC(15:0)	1.19348	8.75	4	482.32	2.17	<0.05	ESI+
LysoPC(14:0/0:0)	1.0021	8.19	3	468.31	1.19	<0.05	ESI+
LysoPE(0:0/16:1(9Z))	1.20375	7.17	2	452.28	1.92	<0.05	ESI+
MG(0:0/18:2(9Z,12Z)/0:0)	1.43675	7.33	5	355.28	−0.25	<0.05	ESI+
MG(17:0/0:0/0:0)	1.0881	7.55	4	367.28	−0.72	<0.05	ESI+
MG(16:1(9Z)/0:0/0:0)	1.10433	9.83	20	329.26	1.08	<0.05	ESI+
Ubiquinone-1	1.39176	6.64	28	251.13	−0.21	<0.05	ESI+
Vitamin D3	1.23479	7.48	14	385.34	2.11	<0.05	ESI+
MG(0:0/15:0/0:0)	1.03958	7.04	0	315.25	1.59	<0.05	ESI−
Deoxycholic acid	1.00367	6.86	1	391.29	1.44	<0.05	ESI−
Leukotriene D4	1.0961	7.82	37	495.27	−0.51	<0.05	ESI−
LysoPA(8:0/0:0)	1.10951	4.75	9	297.11	0.67	<0.05	ESI−
Glycerol 3-phosphate	1.2641	1.06	25	206.99	2.01	<0.05	ESI−
LysoPE(0:0/16:0)	1.4543	7.83	1	452.28	1.92	<0.05	ESI−
LysoPE(0:0/18:1(9Z))	1.48044	8.07	1	478.29	2.36	<0.05	ESI−
Corticosterone	2.10136	10.19	23	381.18	2.85	<0.05	ESI−
Auxin a	1.053	5.99	3	327.22	0.98	<0.05	ESI−
**OTHERS**							
Dopamine	1.37056	3.68	9	176.07	0.78	<0.05	ESI+
Vanillic acid	1.22091	2.64	3	169.05	−0.83	<0.05	ESI+
Hippuric acid	1.15937	2.45	21	202.04	−0.18	<0.05	ESI+
4-Aminosalicylic acid	1.66214	0.76	22	176.04	2.55	<0.05	ESI+
Succinic acid	1.52258	14.25	14	119.03	−0.18	<0.05	ESI+
Phosphoglycolic acid	1.55235	14.27	4	156.99	−0.20	<0.05	ESI+
Sphinganine	1.80699	8.51	3	324.29	1.82	<0.05	ESI+
1-Phenylethylamine	1.39625	14.05	1	122.10	−0.21	<0.05	ESI+
Phosphorylcholine	1.04631	2.31	9	206.06	0.77	<0.05	ESI+
Choline	1.6994	0.75	0	104.11	2.99	<0.05	ESI+
Myo-Inositol	1.27085	2.45	28	203.05	−0.19	<0.05	ESI+
D-Mannitol	1.63547	6.35	27	183.08	−0.19	<0.05	ESI+
D-Maltose	1.26031	0.78	4	365.10	2.49	<0.05	ESI+
Phosphohydroxypyruvic acid	1.8193	14.23	2	184.99	−0.19	<0.05	ESI+
Riboflavin	1.32022	2.78	2	377.15	1.06	<0.05	ESI+
L-Tryptophan	2.05113	2.39	8	205.10	1.28	<0.05	ESI+
Niacin	2.32403	0.99	1	124.04	1.87	<0.05	ESI+
Thymine	1.29367	1.14	3	127.05	1.94	<0.05	ESI+
Kynurenic acid	1.21658	2.50	2	190.05	−0.70	<0.05	ESI+
Gentisic acid	1.10152	2.84	10	153.02	−2.32	<0.05	ESI−
Salicylic acid	1.02709	3.74	11	137.02	−2.62	<0.05	ESI−
Homogentisic acid	1.68695	0.89	3	203.01	2.88	<0.05	ESI−
Sphingosine	1.25312	8.62	17	334.25	−1.37	<0.05	ESI−
Indoleacetic acid	1.65101	2.38	13	210.03	−0.51	<0.05	ESI−
2-Acetolactic acid	1.34723	1.84	12	131.03	0.58	<0.05	ESI−
MG(22:2(13Z,16Z)/0:0/0:0)	1.33733	8.24	2	433.33	1.40	<0.05	ESI+

*RT, retention time; fold change, concentrate group vs. forage group. Mass Error in ppm, the difference between a theoretical m/z and an experimentally observed m/z. ESI+, positive ion mode; ESI−, negative ion mode*.

**Figure 6:**
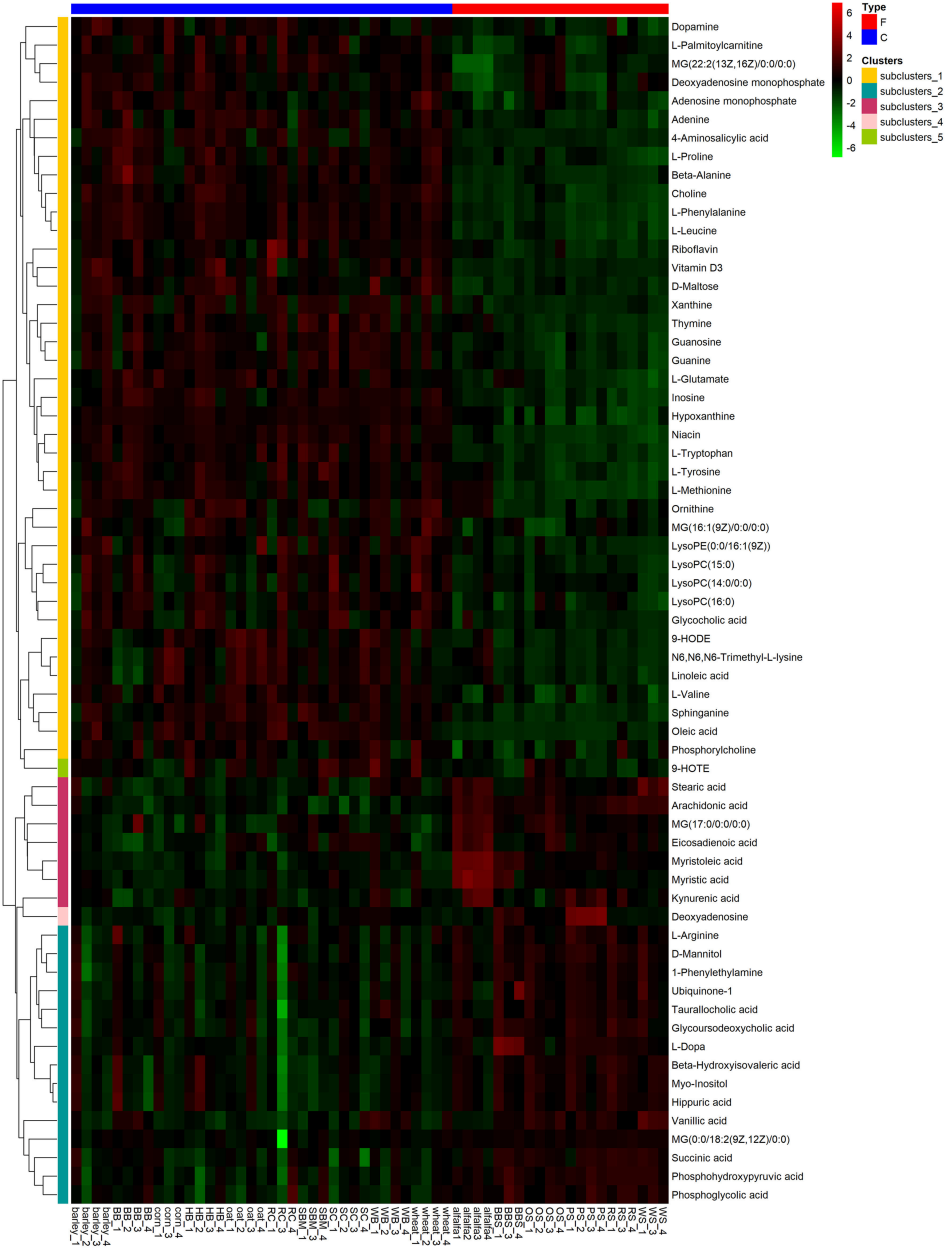
Hierarchical clustering analysis for identification of different metabolites in yak rumen by comparison of the concentrate and forage groups following positive mode ionization. Each column in the figure represents a sample, each row represents a metabolite, and the color indicates the relative amount of metabolites expressed in the group; Red indicates that the metabolite is expressed at high levels, and green indicates lower expression.

**Figure 7:**
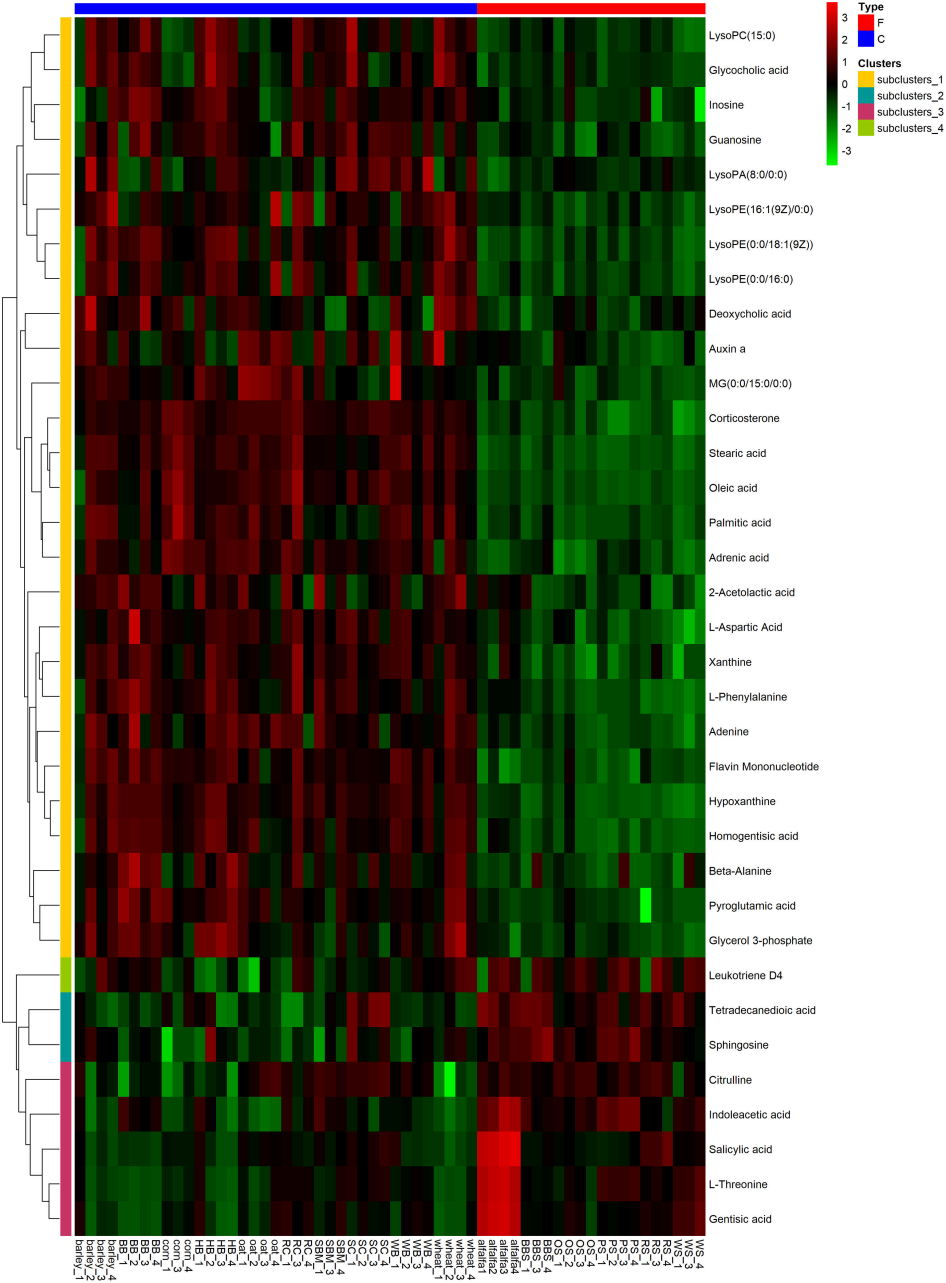
Hierarchical clustering analysis for identification of different metabolites in yak rumen by comparison of the concentrate and forage groups following negative mode ionization. Each column in the figure represents a sample, each row represents a metabolite, and the color indicates the relative amount of metabolites expressed in the group; Red indicates that the metabolite is expressed at high levels, and green indicates lower expression.

**Figure 8:**
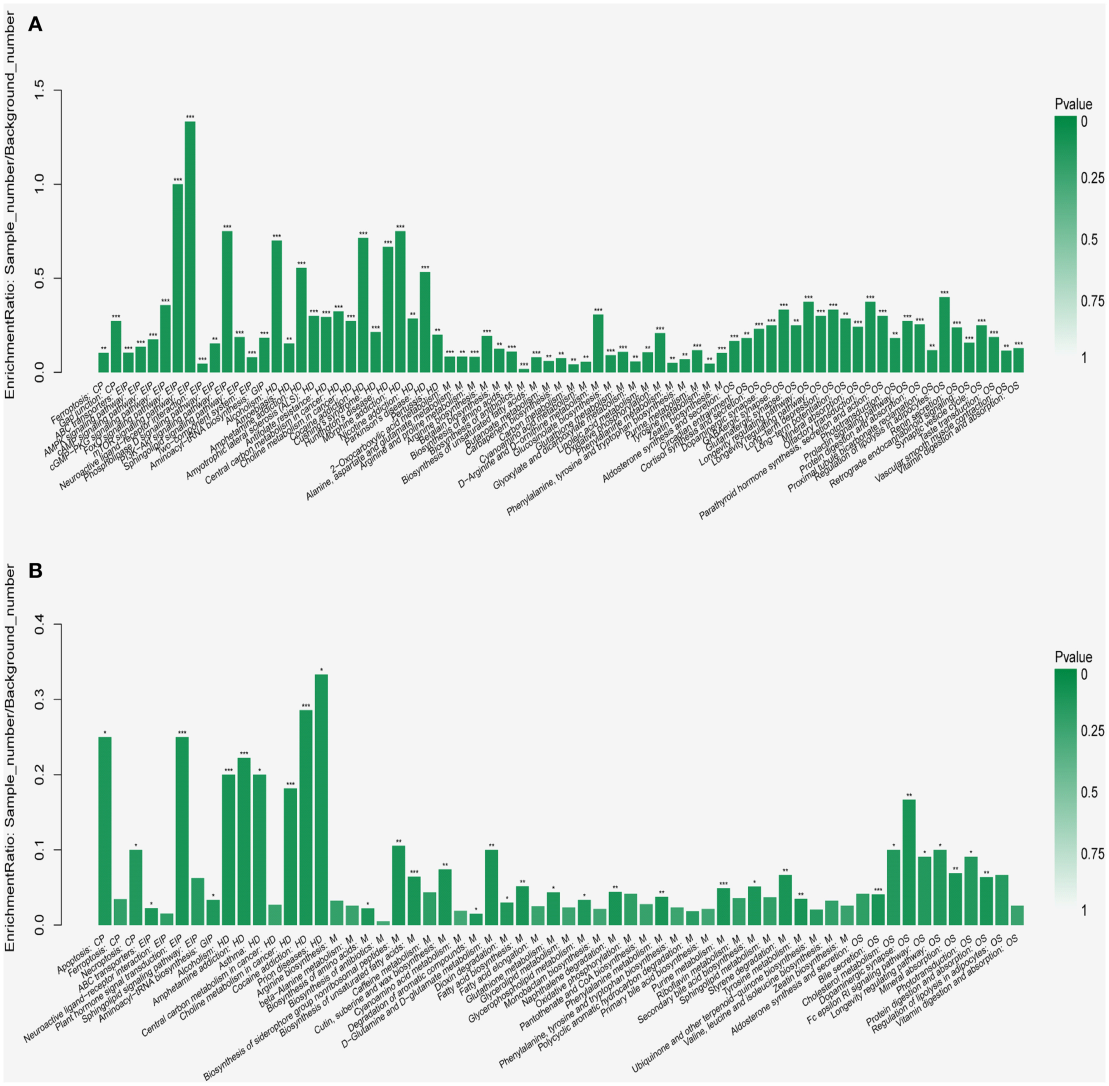
Metabolic pathway enrichment analysis following **(A)** positive and **(B)** negative mode ionization. Overview of metabolites that were enriched in the concentrate group compared to the forage group. CP, EIP, GIP, HD, M, and OS are the class names of the metabolic pathways in the KEGG annotation. CP, Cellular Processes; EIP, Environmental Information Processing; GIP, Genetic Information Processing; HD, Human Diseases; M, Metabolism; OS, Organismal Systems. These were added as suggested (*P < 0.05; **P < 0.01; ***P < 0.001).

To visualize the differences in the yak rumen metabolome associated with the two feed types, we performed the hierarchical clustering analysis (HCA) with a heat map. For the positive ionization data (Figure 6), five distinct clusters were formed among these differential metabolites. It was observed that 40 metabolites, including hypoxanthine, adenine, L-palmitoylcarnitine, 4-aminosalicylic acid, sphinganine, niacin, and L-phenylalanine, were grouped into cluster 1. Cluster 2 included 15 metabolites, such as glycoursodeoxycholic acid, succinic acid, β-hydroxyisovaleric acid, hippuric acid, L-DOPA and phosphohydroxypyruvic acid. Cluster 3 consisted of stearic acid, myristoleic acid, myristic acid, MG (17:0/0:0/0:0), kynurenic acid, eicosadienoic acid and arachidonic acid. Deoxyadenosine and 9-HOTE formed separate groups as cluster 4 and cluster 5, respectively. For the negative ionization data (Figure 7), stearic acid, corticosterone, hypoxanthine, L-phenylalanine, flavin mononucleotide, homogentisic acid, L-aspartic acid and 2-acetolactic acid were grouped in cluster 1. Cluster 2 included tetradecanedioic acid and sphingosine, while other metabolites, including LysoPA (8:0/0:0), salicylic acid, L-threonine, indoleacetic acid, gentisic acid and citrulline, formed cluster 3. And leukotriene D4 clustered separately as cluster 4. The feed type had a significant effect on the rumen metabolome and such differences were clearly observed in the clusters generated in the heatmap plot generated by HCA. For the positive ionization, we observed that cluster 1 and cluster 5 were upregulated in the concentrate group relative to the forage group, whereas clusters 2, 3 and 4 were downregulated in the concentrate group. For the negative ionization analysis (Figure 6), the contents of cluster 1 was increased in the concentrate compared with those in the forage group, while the contents of clusters 2 and 3 were decreased. Furthermore, the enrichment analysis (Figure 8) showed that metabolic pathways, such as protein digestion and absorption, purine metabolism and fatty acid biosynthesis were significantly affected (*P* < 0.05) by changes in the feed type.

Changes in the metabolite profile of a microbial community reflect changes in microbial community dynamics; therefore, we attempted to define relationships between microbial community structure and metabolic function based on microbial and metabolomics data. Correlation analysis of associations between microorganisms and metabolite features provides a comprehensive understanding of the composition and function of microbial communities.

**Figure 9:**
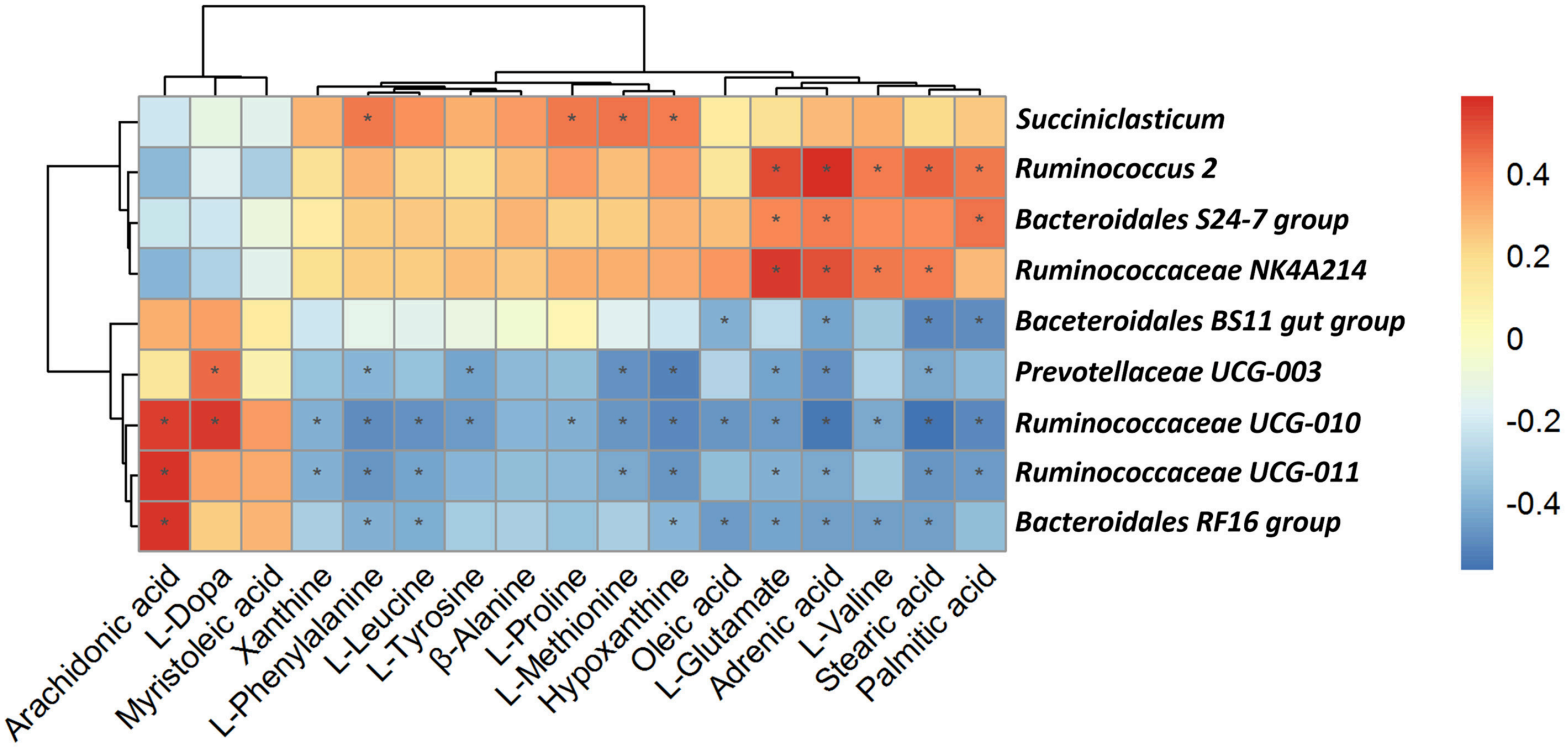
Correlation analysis between genera and metabolite concentrations affected by the feed type. Each row in the graph represents a genus, each column represents a metabolite, and each lattice represents a Pearson correlation coefficient between a component and a metabolite. Red represents a positive correlation, while blue represents a negative correlation. *Significant correlation between the concentrate and forage groups (*P* < 0.05).

As shown in Figure 9, among the bacterial communities with a relatively high abundance at the genus level in the forage group, the genus *Bacteroidales BS11 gut group* was negatively associated with Oleic acid, Adrenic acid, Stearic acid and Palmitic. The genus *Prevotellaceae UCG-003* mainly was negatively associated with L-Phenylalanine, L-Tyrosine, L-Methionine, Hypoxanthine, L-Glutamate, Adrenic acid, Stearic acid and Palmitic acid while was positively associated with L-Dopa. L-Phenylalanine, L-Leucine, Hypoxanthine, L-Glutamate, Adrenic acid and Stearic acid were negatively associated with same three genera which including *Ruminococcaceae UCG-011, Bacteroidales RF16 group* and *Ruminococcaceae UCG-010*. Meanwhile, these three genera were positively associated with Arachidonic acid. On the other hand, the genus *Bacteroidales S24-7 group*, having a higher abundance in the concentrate group, was positively associated with L-Glutamate, Adrenic acid and Palmitic acid. The genus *Ruminococcaceae NK4A214 group*, which was present at higher levels in the concentrate group, was positively associated with L-Glutamate, Adrenic acid, L-Valine and Stearic acid. The genus *Succiniclasticum* was positively associated with a number of metabolites that were upregulated in the concentrate group, including L-Phenylalanine, L-Proline, L-Methionine and Hypoxanthine. And the genus *Ruminococcus 2* was positively associated with L-Glutamate, Adrenic acid, L-Valine, Stearic acid and Palmitic.

## Discussion

Ruminal pH is regulated through behavioral and physiological mechanisms under different feeding regimes (National Academies of Sciences Engineering and Medicine, 2016). In this study, we use the single feedstuff to feed yak individually, which may explain the different values obtained from feeding yak in a total mixed rations (TMR) containing various proportions of forage and concentrates (Xue et al., 2018). Different feeding regimes may result in different rumen internal environment, which indicated that pH values may be different. A significant difference of the NH_3_-N (ammonia nitrogen) which is the source of microbial protein between two groups could be explained that the concentrate group could produce more ruminal microbe crude protein (Zhang J. et al., 2017). Our study reported a higher total VFA production and the proportion of acetate and propionate in the forage group, which was consistent with other studies (Hua et al., 2017; Zhang et al., 2018). However, in mostly cases, those VFA profiles were showed higher level in the concentrate group (Wiener et al., 2003; National Academies of Sciences Engineering and Medicine, 2016). Previous study has reported that VFAs are the end-products of numbers of microbiota and the molar proportions of individual VFAs in the rumen have potential to indicate shifts in bacterial community composition (Saleem et al., 2013). Feed type changes not only modify the available fermentation substrates, but also ruminal environment including pH and VFA profiles, which will affect the metabolic pathways used by the microbes (Ghimire et al., 2017). Thus, we assumed that bacteria changes in the yak rumen might explain the higher total VFA production and the proportion of acetate and propionate in the forage group. In our study, we found that the genus *Ruminococcus 2* was the dominant yak rumen bacteria and it was negatively correlated with acetate and propionate concentrations. Besides, the genus *Bacteroidales BS11 gut group* which were at higher level in the forage group are specialized to active acetate production. Besides, since acetate accounts for a large proportion of total VFA, the increase in acetate content was accompanied by an increase in total VFA content.

In this study, we described the composition of the rumen bacterial community of yak. In accordance with previous studies of ruminants (Leng et al., 2011; Chen et al., 2015; Guo et al., 2015; Xue et al., 2016; Han et al., 2017; Zhou et al., 2017), we found that the core microbiome was dominated by the phyla *Bacteroidetes, Firmicutes*, and *Proteobacteria*. At the genus level, the dominant yak rumen bacteria included *Prevotella 1, Ruminococcus 2, Butyrivibrio 2, Bacteroidales BS11 gut group*, and *Rikenellaceae RC9 gut group*, while *Bacteroidales S24-7 group, Christensenellaceae R-7 group, Prevotellaceae UCG-003* and *Ruminococcaceae NK4A214 group* were also detected. Diversity and richness of the bacterial community are known to be influenced by feed type (McCann et al., 2014; Henderson et al., 2015; Zhang J. et al., 2017). Alpha diversity metrics (Shannon index and Chao1 value) displayed a higher bacterial diversity and richness in the forage group compared to that in the concentrate group, suggesting that different feed types have a direct impact on the yak ruminal microbial composition.

At the genus level, *Bacteroidales S24-7 group* and *Succiniclasticum* were detected in higher abundance in the concentrate group compared to that in the forage group. *Succiniclasticum* has been reported as the main participant in the fermentation of succinate to propionate, which is the most important precursor of glucose in ruminants (van Gylswyk, 1995). Studies have shown that the genera *Bacteroidales S24-7 group*, belonging to the S24-7 family, may be capable of starch utilization (Serino et al., 2012; Anderson et al., 2016). *Ruminococcus 2* was more abundant in yaks fed the concentrate, in the meanwhile, we observed that *Ruminococcus 2* was negatively correlated with acetate and propionate concentrations. These observations were consistent with previous study that there may be resistant starch in the concentrate group which stimulated *Ruminococcus 2* growth probably due to its characteristic amylolytic activity (Ferrario et al., 2017). In the present study, the concentrate group had a higher proportion of the genera *Ruminococcaceae NK4A214 group* which was positively correlated with isobutyrate and isovalerate concentration. Therefore, the lower level of isobutyrate and isovalerate concentration in the forage group could be explained. In contrast, with the exception of the *Bacteroidales S24-7 group, Ruminococcus 2* and *Ruminococcaceae NK4A214 group*, unclassified *Bacteroidales* and *Ruminococcaceae* were present in higher relative abundances in the forage group compared to those in the concentrate group. *Bacteroidales BS11 gut group* are specialized to active hemicellulose monomeric sugars (e.g., xylose, fucose, mannose and rhamnose) fermentation and short-chain fatty acid (e.g., acetate and butyrate) production that are vital for ruminant energy (Solden et al., 2017). *Bacteroidales RF16 group* were more abundant in yaks in the forage group, and other studies have demonstrated the presence of the RF16 family in yak (Xue et al., 2016), beef cattle (Popova et al., 2017) and dairy cattle (Schären et al., 2018); however, the mechanism of RF16 family metabolism is not yet clear. In our study, we found that *Bacteroidales RF16 group* was negatively correlated with isobutyrate and isovalerate concentrations. It is worth exploring the possibility that the relative proportion of *Bacteroidales RF16 group* could effect the production isobutyrate and isovalerate. Studies have provided evidence supporting the involvement of *Ruminococcaceae UCG-011* and *Ruminococcaceae UCG-010* in fiber degradation and ruminal biohydrogenation (Huws et al., 2011; Gagen et al., 2015; Opdahl et al., 2018). In this study, we observed that *Ruminococcaceae UCG-011* and *Ruminococcaceae UCG-010* were negatively correlated with valerate concentrations. It has been reported that bacteria in the rumen could synthesize odd branched-chain fatty acids *de novo* through the elongation of propionate and valerate or the alteration of α-keto acids (Bainbridge et al., 2018). Higher fiber content in the forage group can potentially enhance the fiber degradation through the action of *Ruminococcaceae UCG-011* and *Ruminococcaceae UCG-010*, this is consistent with our findings. So we think this observation may promotes the synthesis of odd branched-chain fatty acids through the valerate. Hence, the lower valerate concentrations in the forage group can be explained. In addition, the genus *Prevotellaceae UCG-003* belongs to the *Prevotellaceae* family, within which the dominant genus *Prevotella* had been reported to represent a group of bacteria that are capable of reducing nitrogen losses, producing succinic and acetic acids as the major fermentation end-products of glucose metabolism and improving the utilization of the forage feed types (Morotomi et al., 2009; Purushe et al., 2010). Thus, it makes sense that the genus *Prevotellaceae UCG-003* presented a higher level in the forage group. In the present study, we revealed that *Prevotellaceae UCG-003* was negatively correlated with valerate and isovalerate concentrations. Previously studies have been reported that branched-chain volatile fatty acids such as isobutyrate and isovalerate are important growth factors for some ruminal bacteria (Allison, 1969) and most species of bacteria could get carbon skeletons from branched-chain volatile fatty acids to use the NH_3_ as a only source of growth (Nolan, 1993). In the present study, we observed the lower level of the isobutyrate and isovalerate concentrations in the forage group and revealed that *Prevotellaceae UCG-003* was negatively correlated with isovalerate. This observation may have been the result of the utilization of branched-chain VFA by the genus *Prevotellaceae UCG-003*. Besides, it has been reported that valerate is glucogenic because it could be used by bacteria and is efficiently metabolize into acetyl-CoA and a propionyl-CoA by β-oxidation (Kristensen, 2007; Lean et al., 2013). Thus, the involvement of the genus *Prevotellaceae UCG-003* in the glucose metabolism could explain the lower level of valerate concentrations in the forage group.

Our metabolome data revealed that feed type alters the concentrations of ruminal metabolites in the rumen, and indicate that ruminal metabolism might be linked with the ruminal microbiota activities. We found that the feed type significantly altered the concentration of most metabolites associated with protein digestion and absorption as well as biosynthesis of amino acids. In the rumen, amino acids, which are key precursors for the synthesis of proteins and polypeptides, regulate some metabolic pathways and are mainly derived from the degradation of dietary proteins and microproteins by the ruminal microbiota (Mariz et al., 2018). In the concentrate group, we observed increased levels of L-phenylalanine and L-tyrosine, both of which have specific characteristics that make them useful as markers of protein metabolism. Furthermore, certain species of bacteria have the ability to generate L-tyrosine from L-phenylalanine under appropriate conditions (Khan et al., 1999; Matthews, 2007). Specifically, L-leucine and L-valine were upregulated in the concentrate group. Previous studies showed that both L-leucine and L-valine tended to decrease the level of acetic, propionic, and butyric acids in the rumen and were extensively metabolized, yielding large quantities of isovaleric and isobutyric acids in the rumen as potential ketogenic and glucogenic substances (Menahan and Schultz, 1964). In addition, previous studies indicated that the proposed intermediates in the metabolism of L-leucine and L-valine are cellulolytically active in rumen microorganisms *in vitro* (Dehority et al., 1958). L-Glutamine is extensively degraded into L-glutamate through hydrolysis mediated by the ruminal microbes of adult steers. L-Glutamate affects microbial growth and efficiency by acting as a potential inhibitor of the utilization of amino acids by ruminal bacteria, while L-glutamate appears to have a greater effect on increasing non-ammonia, non-microbial nitrogen flow than on decreasing microbial nitrogen (Dann et al., 2006; Gilbreath et al., 2017). Furthermore, it has been reported that glutamate is the largest contributor to tricarboxylic acid cycle intermediate fluxes and dietary composition alters L-glutamate catabolism via the tricarboxylic acid cycle in ruminal epithelial cells (El-Kadi et al., 2009). In this study, we observed higher levels of L-methionine in the concentrate than in the forage group. L-methionine is a precursor to other sulfur-containing amino acids and the essential and limiting amino acid for ruminant growth as well as production. In most rumen microorganisms, a significant amount of L-methionine is synthesized from glucose, inorganic sulfur and homocysteine. In addition, L-methionine also plays important roles in rumen bacteria cell protein synthesis and regulation of the mucosal response to antigens (Or-Rashid et al., 2001; Sakkas et al., 2012; Firkins et al., 2015; Mariz et al., 2018). In the present study, we also observed increased levels of L-DOPA, which is a non-protein amino acid with strong allelopathic activity and a precursor of dopamine and sulpiride as well as a D2-type DA receptor blocker. L-DOPA is produced by plants, especially broad beans, and influences the secretion of growth hormone and prolactin in steers (Soares et al., 2014; Kasuya et al., 2017). There are some reports that ruminal tyrosine hydroxylase may catalyze the conversion of tyrosine to L-DOPA, while bacteria, which are the main fermenters of L-DOPA, use the nitrogen in the L-DOPA as a nutrient source in the rumen. In addition, decreasing the concentration of readily degradable components, such as starch and non-amylase-treated neutral detergent fiber (non-aNDF) carbohydrates, was shown to cause an increase in L-DOPA concentrations (Chikagwa-Malunga et al., 2009). Notably, among the amino acids that were upregulated in the concentrate group, the functional amino acid L-proline can be synthesized from arginine and glutamine. L-proline serves as a major amino acid for maintaining cell structure and function, as well as functioning as an important regulator of cell metabolism and physiology. In addition, L-proline plays important roles in protein synthesis and structure, metabolism and nutrition, as well as anti-oxidative reactions in wounds and immune responses (Wu et al., 2011). It has been reported that L-proline levels increase in the yak rumen with increasing levels of dietary concentrate (Zhang R. et al., 2017). β-Alanine, which has been proposed as an intermediate in the formation of acrylamide and acetonitrile or as a direct precursor of poly-β-alanine, is normally metabolized into acetic acid. Higher concentrations of β-alanine are related to larger amounts of starch and readily available carbohydrates, indicating that the conversion efficiency from volatile fatty acids to carbohydrates is decreased (Ametaj et al., 2010; Saleem et al., 2012; Sanchez et al., 2017; Tao et al., 2017).

It is noteworthy that we observed alterations in the concentrations of several metabolites associated with purine metabolism. In the rumen, dietary nitrogen from the feed is degraded and reused by the microbial population for the synthesis of microbial nucleic acids. These nucleic acids are first derived from, and degraded to, purine nucleosides (e.g., inosine and guanosine) through de novo synthesis and the salvage pathway, then to purine bases (e.g., xanthine, hypoxanthine, guanine, and adenine) by enzymatic action, and finally, to purine degradation products (Fujihara and Shem, 2011; Stentoft et al., 2015). In the present study, we found that higher levels of these metabolites in the concentrate group, and in accordance with other studies, the high concentrate diet caused increased levels of xanthine and hypoxanthine, which are nucleic acid degradation products of rumen bacteria. Furthermore, purine is catabolized through a few intermediates to hypoxanthine, which is converted to xanthine; thus, xanthine and hypoxanthine are regarded as biomarkers of microbial protein synthesis (Mcallan and Smith, 1973; Ametaj et al., 2010; Zhang R. et al., 2017). These findings suggest that the concentrate feed type contributes to purine metabolism in the rumen.

Additionally, the altered concentrations of six fatty acids (stearic, oleic, myristoleic, arachidonic, palmitic and adrenic acids) in the rumen suggest that the feed type has an effect on fatty acid biosynthesis and metabolism. Ruminal long-chain fatty acid concentrations are indicative of active lipolysis, biohydrogenation and microbial fatty acid synthesis in the rumen, and the fatty acids are of dietary origin as well as the result of rumen microbial biohydrogenation of dietary lipids (Or-Rashid et al., 2007; Moate et al., 2008; Buccioni et al., 2012). Among the upregulated fatty acids in the concentrate group, stearic acid is the end-product of the biohydrogenation of oleic, linoleic, and linolenic acids. Previous studies demonstrated that the concentrate to forage ratio plays an important role in the accumulation of biohydrogenation intermediates, while, endogenous plant factors involved in ruminal lipid metabolism, such as active plant metabolites that are recognized as modifiers of biohydrogenation of fatty acids, increase stearic acid in the rumen (Mele et al., 2006; Lee and Jenkins, 2011; Buccioni et al., 2012; Yanza et al., 2018). Oleic acid, the product of dietary lipids ingested by ruminants, is hydrogenated by bacteria in the rumen to release free fatty acids, which then are available for the formation of hydroxystearic and ketostearic acids from oleic acid (Jenkins et al., 2006; Hudson et al., 2010; Wu et al., 2016). Palmitic acid, a dietary source of 16-carbon saturated fatty acids, can be used as an energy source for milk production and replenishing BW loss during periods of negative energy balance. In addition, adrenic acid is formed by arachidonic acid chain elongation or elongation and desaturation of the essential fatty acid linoleic acid. Adrenic acid can also be converted to arachidonic acid by β-oxidation; however, the role of adrenic acid and its metabolites in the rumen remains to be clarified. Arachidonic acid, which is a ubiquitous component of every mammalian cell, is not only a tetra-unsaturated fatty acid important for normal cellular membrane fluidity, but also plays other critical biochemical roles, including being the direct precursor of bioactive lipid mediators, such as prostaglandin and leukotrienes (Martin et al., 2016). In addition, arachidonic acid serves as a specific and sensitive plasma biomarker of average daily gain (ADG) in steers (Artegoitia et al., 2017). In this study, we found a higher concentration of adrenic acid in the forage group compared with that in the concentrate group. It has been reported that myristoleic acid can be extracted from the some plants or biosynthesized from myristic acid by the enzyme delta-9 desaturase. Myristoylation is an important post-translational modification required for the transfer of some proteins from the cytosol to the plasma membrane for signal transduction or activation (Kwon et al., 2015; Lapina et al., 2016). However, the metabolism of adrenic acid in the rumen remains to be elucidated.

Throughout the study, there was a utilization or productive association between the composition of the yak rumen bacteria and the rumen metabolome. Furthermore, feed type was shown to have a direct influence on the bacterial community and metabolites in the rumen. Accumulating evidence indicates that phenotypic traits of animals are driven by rumen microbes, while the concentrations of metabolites in the rumen influence the rumen bacterial functions (Mao et al., 2015; Morgavi et al., 2015; Hua et al., 2017; Schären et al., 2018). Overall, these changes and relationships reveal important features associated with the provision of different feed types to yak, and that alterations in the rumen microbial composition and metabolic profiles could be a major factor that influences yak production.

In summary, the present study involved a combination of microbiome and metabolomics analyses of associations between the specific bacterial genera and metabolites in the yak rumen that were significantly influenced by feed type. Integrative information about the interactions between certain metabolites and microbial composition in the yak rumen could provide a better understanding of ruminal metabolites and microbial functions that contribute to the development of modern yak husbandry strategies. Furthermore, understanding the causes and mechanisms driving the interactions among ruminal bacteria and rumen metabolism merits further investigation.

## Author Contributions

The study was designed by SL, SC, QM, and ZZ. Sample processing was carried out by HW and CL. Data analysis was done by CL. The manuscript was written by CL, and was modified by HW and ZZ. All authors read and approved the final manuscript.

### Conflict of Interest Statement

The authors declare that the research was conducted in the absence of any commercial or financial relationships that could be construed as a potential conflict of interest.
