# Supplementary material for: Dynamic Alterations in Yak Rumen Bacteria Community and Metabolome Characteristics in Response to Feed Type
Source: Front Microbiol. 2019 May 22;10:1116. doi: 10.3389/fmicb.2019.01116 (PMC6538947; doi:10.3389/fmicb.2019.01116)
Supplement: Supplementary file 1 [file Table_1.docx]

**Supplementary Table S1 Feeding regimes of four 4 × 4 Latin square trails**

**Supplementary Table S1-1 1st 4 × 4 Latin square design**

| **Item** | **yak1** | **yak2** | **yak3** | **yak4** |
| --- | --- | --- | --- | --- |
| **Ⅰ** | **soybean meal** | **broad bean** | **rape cake** | **sesame cake** |
| **Ⅱ** | **broad bean** | **rape cake** | **sesame cake** | **soybean meal** |
| **Ⅲ** | **rape cake** | **sesame cake** | **soybean meal** | **broad bean** |
| **Ⅳ** | **sesame cake** | **soybean meal** | **broad bean** | **rape cake** |

**Supplementary Table S1-2 2nd 4 × 4 Latin square design**

| **Item** | **yak5** | **yak6** | **yak7** | **yak8** |
| --- | --- | --- | --- | --- |
| **Ⅰ** | **oat** | **hulless barley** | **corn** | **barley** |
| **Ⅱ** | **hulless barley** | **corn** | **barley** | **oat** |
| **Ⅲ** | **corn** | **barley** | **oat** | **hulless barley** |
| **Ⅳ** | **barley** | **oat** | **hulless barley** | **corn** |

**Supplementary Table S1-3 3rd 4 × 4 Latin square design**

| **Item** | **yak9** | **yak10** | **yak11** | **yak12** |
| --- | --- | --- | --- | --- |
| **Ⅰ** | **wheat** | **wheat bran** | **wheat straw** | **pea stem** |
| **Ⅱ** | **wheat bran** | **wheat straw** | **pea stem** | **wheat** |
| **Ⅲ** | **wheat straw** | **pea stem** | **wheat** | **wheat bran** |
| **Ⅳ** | **pea stem** | **wheat** | **wheat bran** | **wheat straw** |

**Supplementary Table S1-4 4th 4 × 4 Latin square design**

| **Item** | **yak13** | **yak14** | **yak15** | **yak16** |
| --- | --- | --- | --- | --- |
| **Ⅰ** | **broad bean stem** | **rapeseed straw** | **oat straw** | **alfalfa** |
| **Ⅱ** | **rapeseed straw** | **oat straw** | **alfalfa** | **broad bean stem** |
| **Ⅲ** | **oat straw** | **alfalfa** | **broad bean stem** | **rapeseed straw** |
| **Ⅳ** | **alfalfa** | **broad bean stem** | **rapeseed straw** | **oat straw** |

yak1, yak2, yak3…… yak 16: sixteen different yaks in the 4 × 4 Latin square design.

Ⅰ, Ⅱ, Ⅲ, Ⅳ: four phases in the 4 × 4 Latin square design.
