# Supplementary material for: Dynamic Alterations in Yak Rumen Bacteria Community and Metabolome Characteristics in Response to Feed Type
Source: Front Microbiol. 2019 May 22;10:1116. doi: 10.3389/fmicb.2019.01116 (PMC6538947; doi:10.3389/fmicb.2019.01116)
Supplement: Supplementary file 2 [file Table_2.doc]

**Supplementary Table S2** Ingredient and Chemical compositions of 16 different feedstuffs diets.

| **Item** | **Concentrate Group (C)** | | | | | | | | | |  | **Forage Group (F)** | | | | | |
| --- | --- | --- | --- | --- | --- | --- | --- | --- | --- | --- | --- | --- | --- | --- | --- | --- | --- |
|  | **soybean meal** | **broad bean** | **rape cake** | **sesame cake** | **oat** | **hulless barley** | **corn** | **barley** | **wheat** | **wheat bran** |  | **wheat straw** | **pea stem** | **broad bean stem** | **rapeseed straw** | **oat straw** | **alfalfa** |
| **Ingredient^1^, % of DM** | | | | | | | | | | | | | | | | | |
| soybean meal | 70 | - | - | - | - | - | - | - | - | - |  | - | - | - | - | - | - |
| broad bean | - | 70 | - | - | - | - | - | - | - | - |  | - | - | - | - | - | - |
| rape cake | - | - | 70 | - | - | - | - | - | - | - |  | - | - | - | - | - | - |
| sesame cake | - | - | - | 70 | - | - | - | - | - | - |  | - | - | - | - | - | - |
| oat | - | - | - | - | 70 | - | - | - | - | - |  | - | - | - | - | - | - |
| hulless barley | - | - | - | - | - | 70 | - | - | - | - |  | - | - | - | - | - | - |
| corn | - | - | - | - | - | - | 70 | - | - | - |  | - | - | - | - | - | - |
| barley | - | - | - | - | - | - | - | 70 | - | - |  | - | - | - | - | - | - |
| wheat | - | - | - | - | - | - | - | - | 70 | - |  | - | - | - | - | - | - |
| wheat bran | - | - | - | - | - | - | - | - | - | 70 |  | - | - | - | - | - | - |
| wheat straw | - | - | - | - | - | - | - | - | - | - |  | 100 | - | - | - | - | - |
| pea stem | - | - | - | - | - | - | - | - | - | - |  | - | 100 | - | - | - | - |
| broad bean stem | - | - | - | - | - | - | - | - | - | - |  | - | - | 100 | - | - | - |
| rapeseed straw | - | - | - | - | - | - | - | - | - | - |  | - | - | - | 100 | - | - |
| oat straw | 30 | 30 | 30 | 30 | 30 | 30 | 30 | 30 | 30 | 30 |  | - | - | - | - | 100 | - |
| alfalfa | - | - | - | - | - | - | - | - | - | - |  | - | - | - | - | - | 100 |
| **Chemical Compositions^2^, % of DM** | | | | | | | | | | | | | | | | | |
| DM | 93.52 | 91.34 | 92.64 | 93.00 | 92.61 | 89.43 | 93.31 | 91.43 | 91.42 | 90.51 |  | 95.52 | 95.06 | 94.5 | 94.69 | 94.74 | 94.98 |
| Ash | 6.11 | 4.64 | 8.27 | 6.05 | 4.60 | 3.64 | 4.19 | 3.77 | 4.42 | 5.67 |  | 5.59 | 6.96 | 9.37 | 6.29 | 7.77 | 13.31 |
| CP | 24.82 | 17.98 | 24.11 | 26.48 | 11.03 | 11.22 | 10.14 | 10.07 | 12.02 | 14.68 |  | 5.74 | 7.95 | 9.39 | 2.44 | 7.75 | 16.01 |
| EE | 2.27 | 1.27 | 5.79 | 3.14 | 1.73 | 1.53 | 6.63 | 1.69 | 2.27 | 2.12 |  | 1.06 | 3.12 | 0.25 | 1.64 | 1.77 | 1.7 |
| Ca | 0.04 | 0.05 | 0.07 | 0.06 | 0.04 | 0.06 | 0.04 | 0.05 | 0.07 | 0.06 |  | 0.03 | 0.11 | 0.11 | 0.1 | 0.05 | 0.11 |
| P | 0.23 | 0.05 | 0.26 | 0.15 | 0.09 | 0.03 | 0.20 | 0.05 | 0.10 | 0.19 |  | 0.03 | 0.02 | 0.07 | 0.03 | 0.08 | 0.29 |
| CF | 10.66 | 12.92 | 10.80 | 11.61 | 12.42 | 9.10 | 9.56 | 9.83 | 13.10 | 12.32 |  | 36.51 | 45.26 | 41.34 | 44.97 | 26.88 | 25.38 |
| NDF | 28.26 | 29.00 | 41.85 | 52.04 | 34.56 | 27.02 | 29.84 | 24.46 | 33.94 | 37.52 |  | 72.33 | 69.14 | 61.85 | 72.95 | 54.4 | 49.32 |
| ADF | 16.14 | 17.30 | 25.87 | 27.11 | 18.89 | 12.20 | 12.79 | 13.06 | 17.70 | 17.21 |  | 44.95 | 51.53 | 46.82 | 55.1 | 31.36 | 33.56 |
| ADL | 1.25 | 6.21 | 10.40 | 9.49 | 5.39 | 1.16 | 1.12 | 1.21 | 1.72 | 1.97 |  | 6.48 | 9.59 | 8.04 | 11.36 | 3.32 | 4.62 |
| **Energy Content, MJ/kg DM** | | | | | | | | | | | | | | | | | |
| DE | 15 | 14.28 | 15.69 | 15.73 | 13.82 | 12.91 | 14.18 | 15.28 | 13.62 | 13.8 |  | 12.93 | 12.49 | 11.78 | 11.98 | 12.31 | 10.92 |
| ME | 11.29 | 9.25 | 11.87 | 10.91 | 10.3 | 8.64 | 11.54 | 11.74 | 10.32 | 9.24 |  | 8.98 | 10.07 | 9.01 | 9.88 | 9.13 | 7.92 |

1. Contained 2% rumen buffer (NaHCO_3_:MgO = 2:1), 0.5% limestone, and 0.5% salt of feed intake on a DM basis and 30 mg/kg rumensin.
2. DM: Dry Matter; CF: Crude Fiber; EE: Ether Extract; NDF: Neutral Detergent Fiber; ADF: Acid Detergent Fiber; ADL: Acid detergent

Lignin.
