# Supplementary material for: Dynamic Alterations in Yak Rumen Bacteria Community and Metabolome Characteristics in Response to Feed Type
Source: Front Microbiol. 2019 May 22;10:1116. doi: 10.3389/fmicb.2019.01116 (PMC6538947; doi:10.3389/fmicb.2019.01116)
Supplement: Supplementary file 4 [file Data_Sheet_1.doc]

**Supplementary Figures**

**
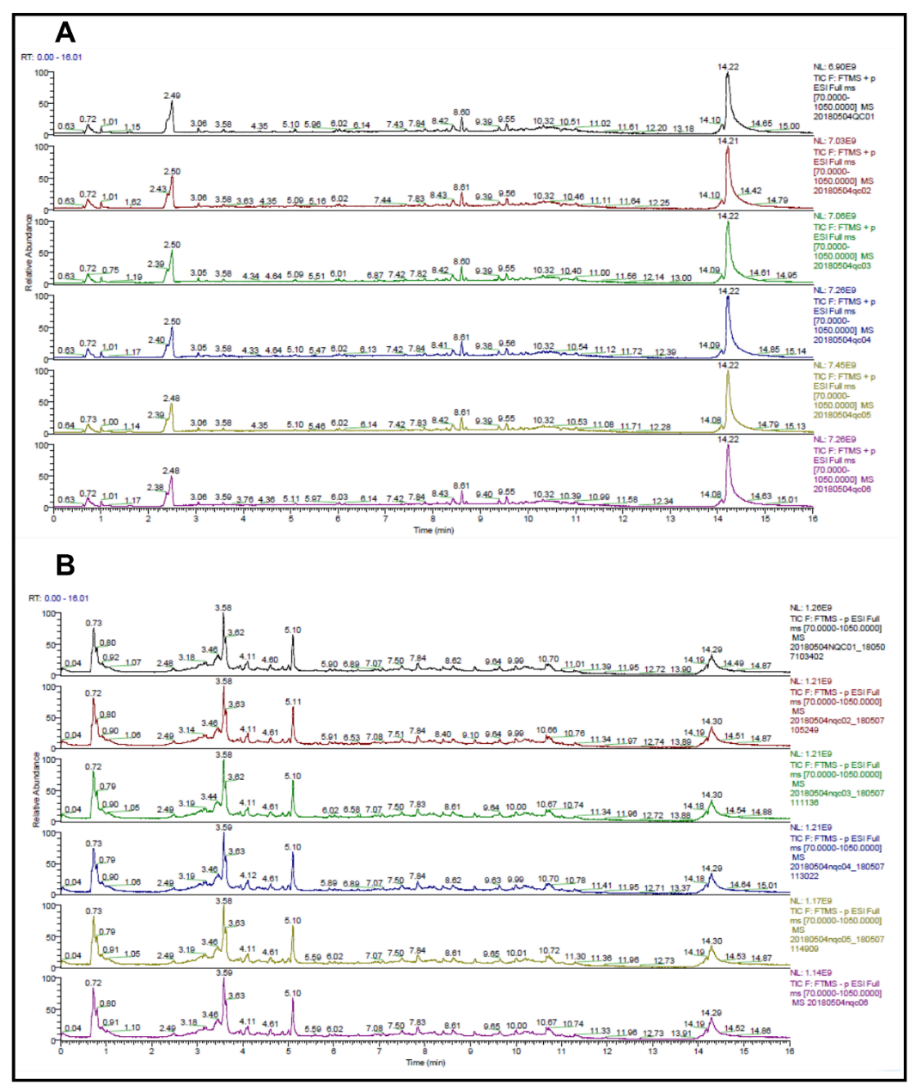
**

**Supplementary Figure S1** LC-MS total ion chromatogram of the QC sample in **(A)** the positive ion mode and **(B)** the negative ion mode.

**
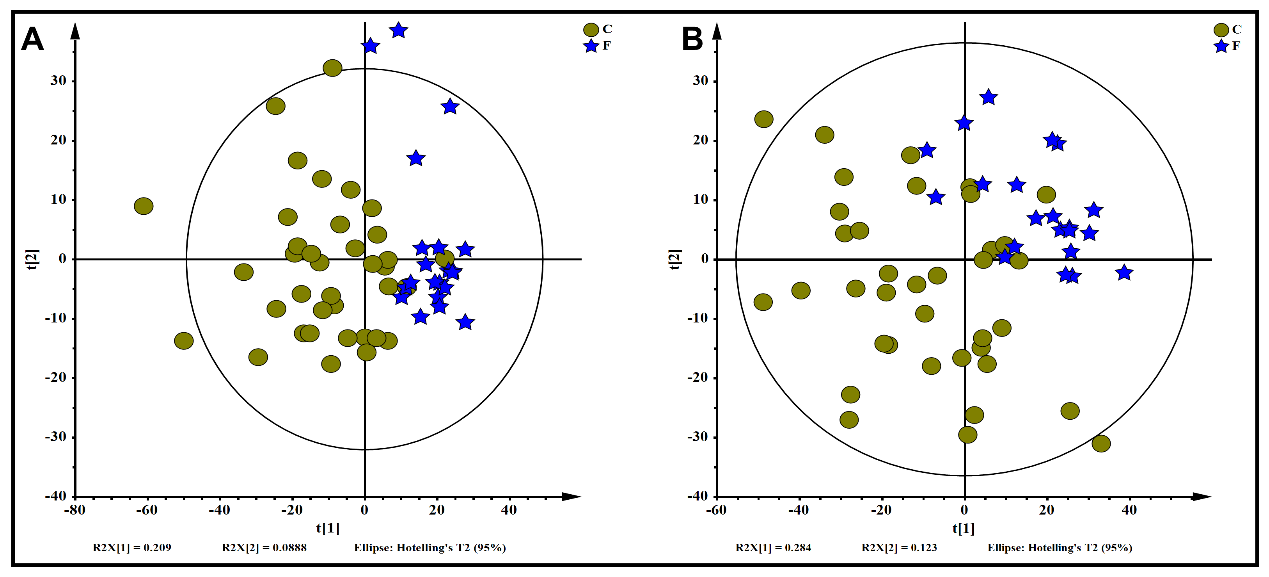
**

**Supplementary Figure S2** Total PCA plot of the yak rumen samples corresponding to different feed groups following **(A)** positive and **(B)** negative mode ionization.
